# Evaluation of TTV replication as a biomarker of immune checkpoint inhibitors efficacy in melanoma patients

**DOI:** 10.1371/journal.pone.0255972

**Published:** 2021-08-09

**Authors:** Rémi Pescarmona, William Mouton, Thierry Walzer, Stéphane Dalle, Anaïs Eberhardt, Karen Brengel-Pesce, Marine Villard, Christine Lombard, Sophie Trouillet-Assant, Sébastien Viel

**Affiliations:** 1 Centre International de Recherche en Infectiologie (CIRI), INSERM U1111, CNRS UMR5308, ENS Lyon, Université Claude Bernard Lyon 1, Lyon, France; 2 Laboratoire d’immunologie, Centre Hospitalier Lyon Sud, Hospices Civils de Lyon, Pierre-Bénite, France; 3 Laboratoire Commun de Recherche, Hospices Civils de Lyon, bioMérieux, Centre Hospitalier Lyon Sud, Pierre-Bénite, France; 4 Virologie et Pathologie Humaine—Virpath Team, Centre International de Recherche en Infectiologie (CIRI), INSERM U1111, CNRS UMR5308, ENS Lyon, Université Claude Bernard Lyon 1, Lyon, France; 5 Université Lyon 1, Lyon, France; 6 Service de dermatologie, Centre Hospitalier Lyon Sud, Hospices Civils de Lyon, Pierre-Bénite, France; Harvard Medical School, Brigham and Women’s Hospital, UNITED STATES

## Abstract

Torque Teno Virus (TTV) is a small, non-enveloped, single-stranded and circular DNA virus that infects the majority of the population worldwide. Increased levels of plasma TTV viral load have been observed in various situations of immune deficiency or dysregulation, and several studies have suggested that TTV levels may be inversely correlated with immune competence. The measurement of TTV viremia by qPCR has been proposed as a potential biomarker for the follow-up of functional immune competence in immunosuppressed individuals, particularly hematopoietic stem cell transplant recipients. We hypothesized that TTV viral load could be used as a prognostic marker of immune checkpoint inhibitor (ICI) efficacy, and therefore investigated the TTV viral load in melanoma patients treated with nivolumab or pembrolizumab before and after 6 months of treatment. In the present study, TTV viral load was not different in melanoma patients before anti-PD-1 introduction compared to healthy volunteers, was not modified by ICI treatment and did not allowed to distinguish patients with treatment-sensitive tumor from patients with treatment-resistant tumor.

## 1. Introduction

Torque Tenuis Virus (TTV), formerly called Transfusion Transmitted Virus was first described in 1997 [1]. It belongs to the *Anelloviridae* family that includes *Alphatorquevirus* genus and represents about 70% of the human virome [2]. It is a small, non-enveloped, single-stranded and circular DNA virus that infects human very early in life [3]. Probably more than 65% of the population worldwide is chronically infected by this virus [4], even if the immune system can control virus replication, which results in low or undetectable viral load.

Although no clinical diseases have been related to TTV infection [3], increased levels of plasma TTV viral load have been observed in various situations of immune deficiency or dysregulation (sepsis, HIV infection, cancer, and autologous or allogeneic hematopoietic stem cell transplantation) [5–8]. Several studies have suggested that TTV levels may be inversely correlated with immune competence [9], as TTV replication is under the control of T lymphocytes [10]. Based on this observation, the measurement of TTV viremia by qPCR has been proposed as a biomarker for the follow-up of functional immune competence in immunosuppressed individuals, particularly hematopoietic stem cell transplant recipients [11–16]. TTV levels were described as relevant biomarker to predict the risk of microbial infections or graft versus host disease, and guide the administration of immunosuppressive therapy or antimicrobial prophylaxis after hematopoietic stem cell transplantation.

T lymphocytes are essential players of cancer immunosurveillance. However, they may present an exhausted phenotype characterized by poor functionality and high expression of “immune checkpoint” receptors such as PD-1 and CTLA-4 when infiltrating tumors. PD-1 and CTLA-4 binding to their ligands, respectively PD-L1 and CD80/CD86, expressed by tumor and other cells, leads to functional exhaustion [17] and impairs anti-tumor immunity. Immune checkpoint inhibitors [ICI] are monoclonal antibodies that were developed to block these ligand/receptor interactions and thus restore T lymphocyte-based immunity and improve clinical outcomes. They were first approved for melanoma, non-small-cell lung cancer and kidney cancer and they have now been shown to provide a survival advantage in many other types of cancer. However, despite promising results in different studies [18–21], only a fraction of patients treated by ICI have clinical benefit whereas many patients experience adverse events or become resistant [22, 23]. In recent years, significant efforts have been undertaken to identify predictive markers of response/toxicity, exploring many immunological and genetic markers. The most widely studied is the expression of PD-L1 on tumor cells or in tumor infiltrating lymphocytes in patients with lung cancer [24–26], and it appears that patients with high PD-L1 expression have a better response rate to PD-1/PD-L1 therapy [27, 28]. Genomic biomarkers, such as tumor mutation burden, could also be good predictive biomarkers of efficacy [29–31]; patients with high burden of tumor mutations deriving greater benefits of the treatment. Similarly, transcriptomic analysis has found a tumor transcriptional signature of poor prognosis [32] or good prognosis; the latter includes high expression of TH1 genes including those coding for IFNγ and PD-L1 [33]. Furthermore, an evaluation of the tumor micro-environment particularly a description of the major phenotype of tumor infiltrating lymphocytes, seems to be very useful to guide ICI treatment [34]. However, almost all biomarkers that have been used to assess ICI biotherapy response suffer from a lack of sensitivity or specificity. There is therefore still a great need for new sensitive and specific predictive biomarkers of ICI efficacy.

In the present study, we hypothesized that TTV viral load could be used as a prognostic marker of ICI efficacy. We investigated this biomarker in a population of melanoma patients, which was described as a condition of immunosuppression [35, 36]: TTV viral load was measured in patients with treatment-sensitive tumor and patients with treatment-resistant tumor before and after 6 months of treatment with anti-PD-1 monoclonal antibodies [nivolumab or pembrolizumab. It was found that TTV viral load was not different in melanoma patients compared to healthy volunteers (HV) before anti-PD-1 introduction. In addition, TTV viral load was neither modified by ICI treatment or allowed to distinguish patients with treatment-sensitive tumor from patients with treatment-resistant tumor.

## 2. Materials and methods

### Study population

This retrospective single-center study included 43 patients [Table 1] treated with anti-PD-1 (nivolumab or pembrolizumab) for metastatic melanoma in the dermatology department of the university hospital (Lyon Sud, Hospices Civils de Lyon, Lyon, France) between December 2013 and April 2019. Written informed consent was obtained from participants ant the study was approved by a regional review board (*Comité de Protection des Personnes Ile de France XI*, Saint-Germain-en-Laye, France, number 12027) and is registered in ClinicalTrial.gov (MelBase, NCT02828202). Each patient was examined prior to the first injection of anti-PD-1 (V1), which was the first line of treatment, and after 6 months of treatment (V2) after which they were classified as either patients with treatment-sensitive tumor (complete or partial response) or patients with treatment-resistant tumor. Concomitantly, 43 age/sex-matched HV from donors to the national blood service (*Etablissement Français du Sang*, EFS) were recruited. According to the EFS standardized procedures for blood donation, and in accordance with the articles R.1243–49 and following ones of the French public health code, written non-opposition for the use of donated blood for research purposes was obtained from healthy individuals. The age and sex of blood donors were forwarded anonymously to the research laboratory. Regulatory authorizations for the handling and conservation of these samples were obtained from the regional ethics committee (*Comité de Protection des Personnes Sud-Est II*, Bron, France) and the French ministry for research (*Ministère de l’Enseignement supérieur de la Recherche et de l’Innovation*, Paris, France).

**Table 1:** Patient characteristics.

	All patients	Treatment-sensitive tumor	Treatment-resistant tumor	Healthy volunteers
Patients (n)	43	18	25	43
Sex (n (%))				
Male	27 (62.8)	9 (50.0)	18 (72.0)	30 (69.8)
Female	16 (37.2)	9 (50.0)	7 (28.0)	13 (30.2)
Age (median years (range))	66 (25–84)	62 (39–81)	69 (25–84)	52 (28–67)
Treatment (n (%))				
Nivolumab	16 (37.2)	6 (33.3)	10 (40.0)	-
Pembrolizumab	27 (62.8)	12 (66.7)	15 (60.0)	-
Stage of disease (n (%))				
IV	37 (86.0)	16 (88.9)	21 (84.0)	-
IIIc	1 (2.3)	-	1 (4.0)	-
IIIb	4 (9.3)	2 (11.1)	2 (8.0)	-
NR	1 (2.3)	-	1 (4.0)	-
Mutations (n (%))				
BRAF	14 (32.6)	6 (33.3)	8 (32.0)	-
KRAS	6 (14.0)	2 (11.1)	4 (16.0)	-
Undetermined	2 (4.7)	-	2 (8.0)	-
Brain meets (n (%))	8 (18.6)	4 (22.2)	4 (16.0)	-

### Plasma collection

Whole blood was collected in EDTA tubes at V1 and V2 for each patient and centrifuged at 2000g for 10 minutes at room temperature. Plasma was then frozen at -80°C until DNA extraction.

### TTV DNA extraction and load quantification

Total nucleic acids (elution volume 50μL) were extracted from 200μL of plasma sample using an easyMag extractor (bioMérieux, Marcy-l’Etoile, France) following the manufacturer’s instructions. The presence and viral load of TTV DNA were then determined using the TTV R-GENE® kit (available for research use only, not for diagnostic procedure; Ref#69–030, bioMérieux) as previously described [37]. Real-time PCR amplification was performed according to manufacturer’s instructions on a Stratagene® Mx3005P™ platform (Stratagene, La Jolla, CA, USA).

### Statistical analysis

Log_10_ transformed TTV load format was used for analysis (log_10_ copy/mL). A Mann-Whitney test was used to compare TTV viral load levels between groups and a *p-value* < 0.05 was considered to indicate statistical significance. Data were analyzed and plotted using GraphPad Prism software (version 5; GraphPad software, La Jolla, CA, USA) and R (version 3.5.1).

## 3. Results

On the 43 patients included in the study, 30 (69.8%) had a detectable TTV viral load prior to the initiation of anti-PD-1 therapy (V1). Similarly, 31/43 (72.1%) HV had a quantifiable TTV viral load (Table 1).

**Fig 1:**
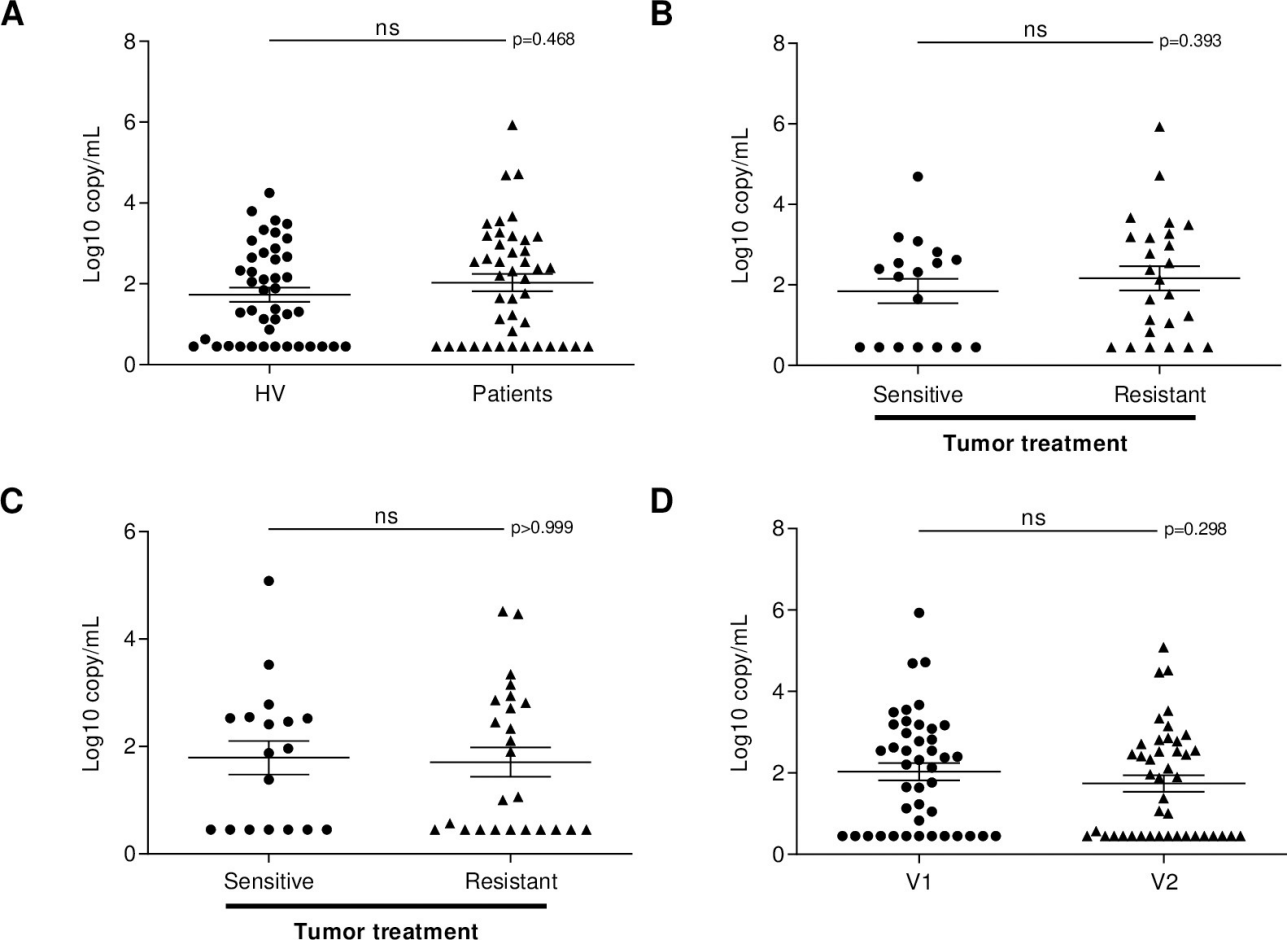
Comparison of TTV viral load in healthy volunteers and melanoma patients before and after 6 months of anti-PD-1 therapy. (A) Torque Teno Virus (TTV) viral load of healthy volunteers (HV) and melanoma patients prior to the initiation of anti-PD-1 therapy (V1). (B) and (C) TTV viral load in the treatment-sensitive tumor and treatment-resistant tumor groups of melanoma patients B) before (V1) and C) 6 months after (V2) the initiation of anti-PD-1 therapy. (D) TTV viral load of the melanoma patients prior to the initiation of anti-PD-1 therapy (V1) and after 6 months of anti-PD-1 therapy (V2). A viral load of 0.45 log_10_ copy/mL as assigned to patients and controls with undetectable viral load which is the lowest limit of detection. ns: non-significant.

The median TTV viral load was not significantly different in melanoma patients (2.20 log_10_ copy/mL) compared to HV (1.38 log_10_ copy/mL, *p* = 0.468; Fig 1A).

There were 18 patients whose tumor responded to treatment and 25 who did not. Before anti-PD-1 initiation (V1), 11 (61.1%) patients with treatment-sensitive tumor and 19 (76.0%) patients with treatment-resistant tumor had a detectable TTV viral load. There was no significant difference in the median TTV viral load between patients with treatment-sensitive tumor (2.26 log_10_ copy/mL) and patients with treatment-resistant tumor (2.13 log_10_ copy/mL; Fig 1B).

After 6 months of treatment (V2), 26/43 (60.4%) of patients had a detectable TTV viral load; 11 of whom were in the treatment-sensitive tumor group and 15 were in the treatment-resistant tumor group. In patients with treatment-sensitive tumor, the TTV load became undetectable for one patient between V1 and V2, while for 6 patients the TTV load remained undetectable, and for one patient the TTV load was detected only at V2. In patients with treatment-resistant tumor, the TTV load became undetectable for 6 patients between V1 and V2, while for 4 patients it remained undetectable, and for 2 patients the TTV load was detected only at V2. In all patients, median viral loads were not significantly different between patients with treatment-sensitive tumor (1.92 log_10_ copy/mL) and patients with treatment-resistant tumor (1.06 log_10_ copy/mL, *p* > 0.999) after 6 months of treatment (Fig 1C).

Finally, there was no significant difference between the median TTV viral load before (2.20 log_10_ copy/mL) and after 6 months of treatment (1.88 log_10_ copy/mL) indicating that the treatment has no impact on TTV replication whether tumor is sensitive or resistant to ICI treatment (Fig 1D).

## 4. Discussion

In the present study, median TTV viral load was not different in untreated melanoma patients (V1) compared to HV, contrary to what it was reported for other immunosuppressive situations [7, 8] and other types of cancer [38].

Moreover, TTV DNA viral load was not different in patients with treatment-sensitive tumor compared to patients with treatment-resistant tumor before anti-PD-1 treatment, and thus did not allow to identify patients’ candidates to ICI treatment. Strikingly, TTV viral load was also not changed after treatment, irrespective of response status. However, ICI, including anti-PD-1, are now well known to unleash T cell responses [39] and a decrease of viral load was therefore expected following immune checkpoint inhibition. The maximum T lymphocyte activity probably occurs during the first weeks after ICI treatment and may be normalized after 6 months, thus it would be interesting to monitor TTV replication earlier than 6 months after antiPD-1 treatment in future studies, for example after 2 or 4 weeks.

It is of note that the results found in melanoma patients contrast with primary lung cancer patients for which TTV DNA viral load is significantly higher compared to HV [40]. This suggests that overall, melanoma patients retain a good T cell function and that the prognostic value of this marker may be greater in cancers where T cell function is more affected.

Finally, about 30% of TTV DNA viral loads were below the limit of detection for patients and HV. If patients with an undetectable viral load were excluded from statistical analysis, the increase of TTV DNA viral load in melanoma patients would have been significant. The development of a more sensitive technique would allow better monitoring of the TTV viral load and its kinetics following ICI treatment.

This study is a proof of concept study and results have to be confirmed on a larger cohort.

## 5. Conclusions

In conclusion, a 6 months treatment with nivolumab or pembrolizumab had no significant impact on TTV replication whether patient tumor was sensitive or resistant to ICI treatment. Consequently, TTV viral load at melanoma diagnosis is not a reliable predictive biomarker of anti-PD-1 efficacy. Before ruling out TTV monitoring as a biomarker of efficacy of ICI treatment, these results have to be confirmed in other types of cancer and with other ICI (ipilimumab, durvalumab, cemiplimab, atezolizumab, avelumab). Moreover, TTV viral load should be monitored earlier than 6 months after treatment initiation.
